# Vegetarian Diet Reduced Gastroesophageal Reflux Disease in a Nationwide Longitudinal Survey in Taiwan

**DOI:** 10.3390/nu16213712

**Published:** 2024-10-30

**Authors:** Jyun-Han Lu, Chun-Chi Tsai, Jia-In Lee, Chih-Yi Lin, Shu-Pin Huang, Jiun-Hung Geng, Chao-Hung Kuo, Szu-Chia Chen

**Affiliations:** 1 Department of Internal Medicine, Kaohsiung Medical University Hospital, Kaohsiung Medical University, Kaohsiung 807378, Taiwan; hank821118@gmail.com; 2 Health Management and Occupational Safety and Health Center, Kaohsiung Municipal Siaogang Hospital, Kaohsiung Medical University Hospital, Kaohsiung Medical University, Kaohsiung 812015, Taiwan; chunchi0303@gmail.com; 3 Department of Psychiatry, Kaohsiung Medical University Hospital, Kaohsiung Medical University, Kaohsiung 807378, Taiwan; u9400039@gmail.com; 4 Administration Management Center, Kaohsiung Municipal Siaogang Hospital, Kaohsiung Medical University Hospital, Kaohsiung Medical University, Kaohsiung 812015, Taiwan; chiyil824@gmail.com; 5 Department of Urology, Kaohsiung Medical University Hospital, Kaohsiung Medical University, Kaohsiung 807378, Taiwan; shpihu@kmu.edu.tw; 6 School of Medicine, College of Medicine, Kaohsiung Medical University, Kaohsiung 807378, Taiwan; kjh88kmu@gmail.com; 7 Department of Urology, Kaohsiung Municipal Siaogang Hospital, Kaohsiung Medical University Hospital, Kaohsiung Medical University, Kaohsiung 812015, Taiwan; 8 Department of Internal Medicine, Kaohsiung Municipal Siaogang Hospital, Kaohsiung Medical University Hospital, Kaohsiung Medical University, Kaohsiung 812015, Taiwan; 9 Division of Gastroenterology, Department of Internal Medicine, Kaohsiung Medical University Hospital, Kaohsiung Medical University, Kaohsiung 807378, Taiwan; 10 Division of Nephrology, Department of Internal Medicine, Kaohsiung Medical University Hospital, Kaohsiung Medical University, Kaohsiung 807378, Taiwan

**Keywords:** vegetarian diet, years and types of vegetarian diet, gastroesophageal reflux disease, Taiwan biobank

## Abstract

Background/Objectives. This large, longitudinal follow-up cohort study aimed to explore how being a vegetarian and related factors impacted the incident gastroesophageal reflux disease (GERD) in a comprehensive Taiwanese cohort. Methods. The study cohort was enrolled from the Taiwan Biobank. Vegetarian status, duration of being a vegetarian, type of vegetarian diet, and whether or not the participants had GERD were recorded from self-reported surveys. Associations between vegetarian status, duration, and type of diet with incident GERD were analyzed with multivariate logistic regression with adjustments for confounding variables. Results. After excluding participants with pre-existing GERD, we included 23,714 participants into the study. Multivariable analysis showed that vegetarian status (current vs. never; hazard ratio [HR], 0.697; 95% confidence interval [CI], 0.546 to 0.889; *p* = 0.004) was significantly inversely associated with incident GERD; conversely, ever being a vegetarian was not associated (*p* = 0.489). In addition, those who had been a vegetarian for 6 years or more had 0.72 times lower risk of GERD compared to those who had never been a vegetarian (HR, 0.717; 95% CI 0.558 to 0.922, *p* = 0.009). No significant differences were observed regarding the type of vegetarian diet with incident GERD. Conclusions. The results showed that following a vegetarian diet was an independent protective factor for incident GERD, with a significant protective effect observed in those who adhered to a vegetarian diet for at least 6 years. Future research is warranted to explore the underlying mechanisms and whether adopting a vegetarian diet can decrease the incidence of GERD.

## 1. Introduction

Gastroesophageal reflux disease (GERD) is characterized by the regurgitation of gastric contents into the esophagus leading to complications or bothersome symptoms [1]. GERD is a global concern, with a prevalence ranging from 2.5% to 7.8% in East Asia and 18.1% to 27.8% in North America [2]. Symptoms associated with GERD include heartburn, acid regurgitation, chest pain, odynophagia, dysphagia, water brash, and globus sensation, and they can significantly impact the quality of life [1,3,4]. Moreover, GERD is a known risk factor for Barrett’s esophagus and has been associated with a higher incidence of esophageal adenocarcinoma [5]. Consequently, evaluating both the risk and protective factors for GERD is crucial.

Dietary choices and weight control play pivotal roles in GERD management [6,7]. Patients often report that eating chocolate, fried and spicy foods can trigger reflux symptoms [8,9]. In addition, many GERD patients avoid citrus fruit juice, carbonated drinks, and other acidic beverages out of concern that they may worsen their symptoms [10]. A vegetarian diet is defined as eating vegetables, nuts, fruits, legumes, and grains while restricting indirect animal foods [11], and has been associated with lower risks of some cancers, obesity, type 2 diabetes, hypertension, and other cardiovascular diseases [12,13,14,15,16,17]. Observational studies in India and Iran suggest that a vegetarian diet can protect against GERD [18,19,20,21]. Studies from Western countries have also demonstrated that plant-based diets are associated with improved gastrointestinal health and a reduced prevalence of reflux symptoms [22,23].

Taiwan presents a unique setting for studying the relationship between vegetarianism and GERD, as the country has a long tradition of plant-based diets, often motivated by religious and ethical beliefs [24]. In Taiwan, vegetarianism is particularly prevalent among certain Buddhist and Taoist communities, with estimates suggesting that approximately 10–13% of the population follows a vegetarian diet [25]. The vegetarian diet in Taiwan often includes a wide variety of soy-based products, such as tofu and tempeh, which are less commonly consumed in other regions with predominantly plant-based diets [26]. Given these cultural and dietary distinctions, Taiwanese vegetarians may exhibit different health outcomes compared to vegetarians in other parts of the world, further justifying the need for region-specific research. Understanding how the distinct characteristics of the Taiwanese vegetarian diet—such as higher consumption of soy products, fermented foods, and specific cooking practices—affect GERD risk is crucial for contextualizing findings in the global literature.

The positive impact of a vegetarian diet on the digestive system may be attributed to its high fiber content and lower consumption of animal fats, which support gastrointestinal transit and may help reduce reflux [27]. In addition, the rich presence of antioxidants such as vitamins C and E, flavonoids, and other bioactive compounds in a vegetarian diet plays a significant role in protecting the esophageal mucosa. These antioxidants help neutralize reactive oxygen species (ROS), reducing oxidative stress on esophageal tissues [27,28,29]. Oxidative stress, a key contributor to the pathogenesis of GERD, triggers inflammation and damages the esophageal lining by promoting lipid peroxidation, protein oxidation, and DNA damage [30,31]. Antioxidants mitigate these harmful processes by scavenging free radicals, thus preventing cellular injury and reducing inflammation in the esophagus [32,33]. Moreover, specific compounds such as flavonoids and carotenoids found in plant-based foods have been shown to inhibit pro-inflammatory cytokine production, further contributing to the protection against esophageal damage [29]. Through these mechanisms, a vegetarian diet may lower the risk of GERD by maintaining mucosal integrity and reducing the esophageal inflammation that exacerbates reflux symptoms.

This study aims to investigate the associations between vegetarian status, duration of vegetarianism, and type of vegetarian diet on the incidence of GERD in a large Taiwanese cohort. We hypothesize that adherence to a vegetarian diet, especially over a longer period, may reduce the incidence of GERD through mechanisms related to improved gastrointestinal function and a reduction in oxidative stress.

## 2. Materials and Methods

### 2.1. Study Participants

All participants in this longitudinal cohort study were enrollees of the Taiwan Biobank (TWB). Established in 2008 as a large-scale community-based research program, the TWB database includes long-term follow-up data from Taiwanese adults aged 30 to 70 years and has been used in previous studies to explore the connections between environmental, clinical, and personal health factors and various diseases.

Eligible participants were required to be of Taiwanese ethnicity, provide written informed consent, and have no history of severe chronic diseases such as cancer at enrollment. Individuals with incomplete baseline data were excluded. Participants were selected based on their willingness to undergo regular follow-ups every two years, during which they provided biospecimens and completed questionnaires regarding lifestyle, medical history, and other health-related factors [34,35,36,37,38,39].

Following initial enrollment, participants attended in-person visits every two years. During these visits, they answered updated questionnaires, provided biospecimens, and underwent physical examinations. The follow-up process was standardized across all participating institutes, and participants received reminders for upcoming visits via phone calls and letters. Data collection was overseen by trained personnel at each follow-up visit. Losses to follow-up were carefully monitored, and reasons for dropout, such as relocation or withdrawal of consent, were documented [34,35,36,37,38,39]. Detailed information on the TWB has been reported previously [34,35,36,37,38,39].

The present study was approved by the IRB of our institute (KMUHIRB-E(I)-20210058, 23 March 2023), and it was performed in accordance with the Helsinki Declaration. An initial cohort of 27,209 participants were screened for eligibility, and those with known underlying GERD (*n* = 3483) and those without age (*n* = 1) and smoking (*n* = 11) data were excluded. The final analysis included 23,714 participants.

The following baseline data were recorded: age, body mass index (BMI; (kg/m^2^), alcohol/smoking status, marital status, physical activity, years of education, current residential situation, systolic/diastolic blood pressures, fluid intake, induced abortion, gravidity, etiology of menopause, hormone therapy, breastfeeding, and parity. We also recorded whether the participants had been diagnosed with gout, hypertension, dyslipidemia, and diabetes mellitus. Systolic/diastolic blood pressures were also documented. We also checked for the presence of chronic kidney disease, defined as an estimated glomerular filtration rate < 60 mL/min/1.73 m^2^ (calculated using the four-variable MDRD equation [40]). Blood pressure was measured using an automated sphygmomanometer, which is clinically validated for accuracy. Each participant’s blood pressure was taken three times, with the average of the second and third readings recorded. Weight and height were measured using a calibrated digital scale and stadiometer, ensuring precise and consistent measurements. All instruments used in this study were calibrated regularly and have been clinically validated for accuracy in both research and clinical settings [34,35,36,37,38,39].

### 2.2. Assessment of Vegetarian Status

The dietary classification was based on self-declaration through a series of structured, closed-ended questions to identify excluded food types. Participants were first asked whether they had ever followed a completely vegetarian diet for at least six months, with responses categorized as “Never”, “Yes, currently”, or “Yes, but not anymore”. Based on their answers, participants were categorized into three groups: Never, Current, and Ever vegetarians. Those in the Ever and Current vegetarian groups were further asked to specify the type of diet—vegan (excludes all animal products), lacto-vegetarian (consumes dairy but no other animal products), ovo-vegetarian (consumes eggs but no other animal products), or lacto-ovo vegetarian (consumes both dairy and eggs)—as well as the duration of being a vegetarian.

### 2.3. Study Outcomes, Incident GERD

We identified whether the participants had GERD through self-reported diagnoses. In the TWB, all participants were reviewed by well-trained personnel who followed standardized procedures. The participants were asked, “Have you been diagnosed with GERD?” at each follow-up visit, and those who replied “Yes” were considered to have developed GERD during follow-up. As any participant with a past GERD diagnosis was excluded, none of the enrolled participants had GERD at baseline.

### 2.4. Statistical Analysis

Data are presented as numbers (%) for categorical variables and as mean (±SD) for continuous variables. Comparisons among the three vegetarian status groups (“never”, “ever”, and “current”) were performed using pairwise Chi-Square tests with Bonferroni correction for categorical variables and one-way Analysis of Variance (ANOVA) with post-hoc tests for continuous variables. Kaplan–Meier survival curves for incident GERD were generated. The time to the occurrence of GERD and risk factors were analyzed with a multivariable Cox proportional hazards model, which was adjusted for systolic/diastolic blood pressures, heart rate, age, BMI, sex, marital status, dependency, ever having smoked tobacco/drunk alcohol, physical activity, educational status, hypertension, diabetes, dyslipidemia, depression, manic depression, alcoholism, schizophrenia, Parkinson’s disease, chronic kidney disease, vegetarian status, duration of being a vegetarian, and type of vegetarian diet. The participants who were vegetarians were considered the reference group, which was at the lowest risk of incident GERD. The adjusted covariates were chosen based on those reaching significant levels in the univariable Cox regression for the risk of GERD. Results were considered significant at *p* < 0.05. SPSS (v20, IBM Inc., Armonk, NY, USA) was used for the statistical analysis.

## 3. Results

### 3.1. Characteristics of the Vegetarian Status Groups at Baseline

Of the 23,714 included participants (mean age 51 ± 10 years; 8398 male, 15,316 female), there were 21,632 (91%) in the non-vegetarian group, 1152 (5%) in the current vegetarian group, and 930 (4%) in the ever vegetarian group (Table 1). The current vegetarian group were older, had lower systolic/diastolic blood pressures, were female predominant, and had lower ever married rate, higher dependency rate, lower smoking and alcohol history, lower exercise habit rate, lower level of education status, lower hypertension rate, and lower dyslipidemia rate than the non-vegetarian group.

### 3.2. Association Between Incident GERD and Vegetarian Status Using Univariable Analysis

A total of 1814 participants (7.6%) developed GERD during a mean follow-up duration of 47 ± 14 months, ranging from 19.6 to 125.4 months. The results of univariable analysis showed that older age, high systolic/diastolic blood pressures, male sex, ever having been married, physical activity, educational status (middle to high vs. elementary school), histories of hypertension, dyslipidemia, depression and manic depression, and vegetarian status (current vs. never; hazard ratio [HR], 0.710; 95% confidence interval [CI], 0.557 to 0.905; *p* = 0.006) were associated with incident GERD; however, the ever vegetarian group was not (*p* = 0.402) (Table 2).

### 3.3. Association Between Incident GERD and Vegetarian Status Using Multivariable Analysis

Multivariable Cox regression analysis results for incident GERD according to vegetarian status are shown in Table 3. A total of 1672 participants (8%) had incident GERD in the non-vegetarian group, compared to 68 participants (6%) in the current vegetarian group, and 74 participants (8%) in the ever vegetarian group. After adjusting for confounders, vegetarian status (current vs. never; HR, 0.697; 95% CI, 0.546 to 0.889; *p* = 0.004) was significantly associated with incident GERD; however, the ever vegetarian group was not (*p* = 0.489).

**Figure 1:**
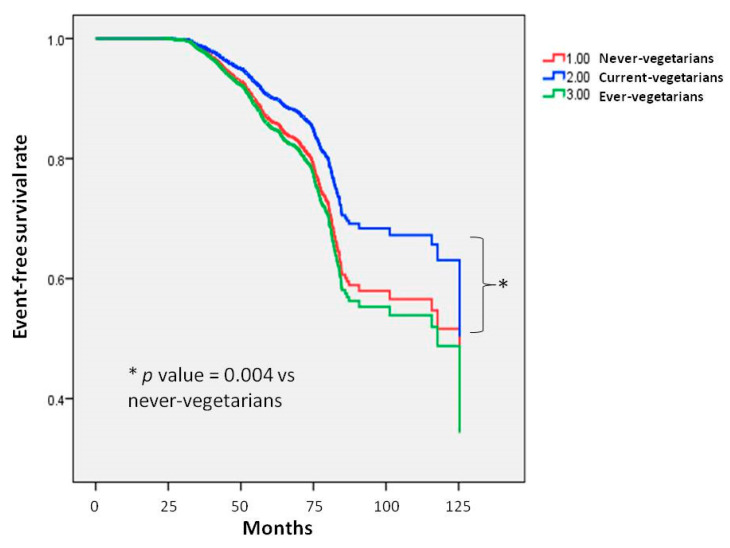
Time to incident GERD was longer in participants currently following a vegetarian diet compared to those who have never followed a vegetarian diet. Kaplan–Meier plot showing the development of incident GERD based on adherence to a vegetarian diet in 23,714 participants with follow-up data.

Kaplan–Meier analysis of incident GERD (log-rank *p* = 0.004) showed that the current vegetarian group had a longer time to incident GERD than the non-vegetarian group (Figure 1).

### 3.4. Association Between Incident GERD and Duration of Being a Vegetarian

To further assess the association between the development of GERD and the duration of being a vegetarian, the participants were categorized into three groups accordingly as 0 years, <6 years, and ≥6 years. Adjusted multivariable Cox regression analysis showed that the ≥6 years group (vs. 0 years; HR, 0.717; 95% CI 0.558 to 0.922, *p* = 0.009) had a lower risk of developing GERD (Table 4).

**Figure 2:**
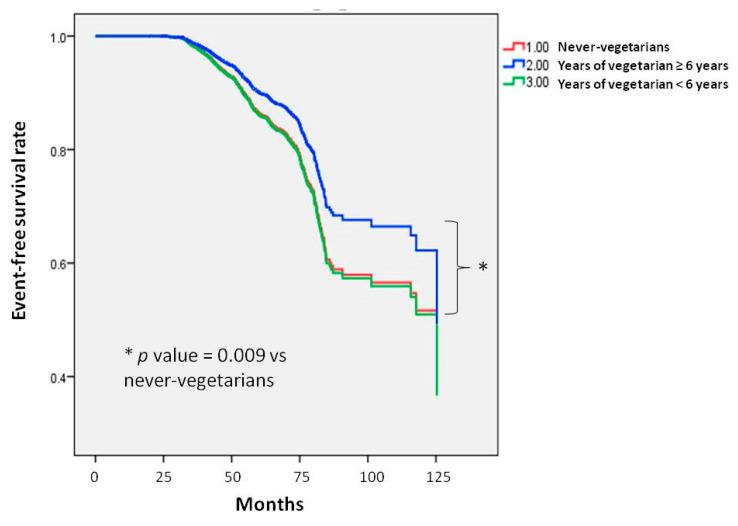
Time to incident GERD was longer in participants following a vegetarian diet for ≥6 years compared to those who have never followed a vegetarian diet. Kaplan–Meier plot showing the development of incident GERD according to years of following a vegetarian diet in 23,714 participants with follow-up data.

Kaplan–Meier analysis of incident GERD (log-rank *p* = 0.009) showed that the time to incident GERD was longer in those who adhered to a vegetarian diet for ≥6 years compared to those who had never adhered to a vegetarian diet (Figure 2). Although no significant decrease was found in the risk of developing GERD between the <6 years group (HR, 1.020; 95% CI 0.812 to 1.281, *p* = 0.865) and non-vegetarian group, the *p* for trend was significant (0.033).

### 3.5. Association Between Incident GERD and Type of Vegetarian Diet

We further categorized the participants into four groups according to whether they followed a vegan, lacto-, ovo-, or lacto-ovo-vegetarian diet to investigate the associations among incident GERD and the type of diet. The results of multivariable Cox regression analysis showed no significant differences in the risk of incident GERD among the four vegetarian diet groups (Table 5).

## 4. Discussion

In this nationally representative longitudinal study, we found that the participants who followed a vegetarian diet were associated with a lower likelihood of being diagnosed with GERD after adjustments for covariates. Furthermore, the participants who had been a vegetarian for 6 years or more also had a lower likelihood of being diagnosed with GERD compared to those who had never been a vegetarian. However, the type of vegetarian diet was not associated with incident GERD.

The World Health Organization reported associations between lower risks of stroke [41,42], cardiovascular diseases [42,43,44,45,46], diabetes [47], and colorectal cancer [48,49] with a plant-based diet. Our study further showed that following a vegetarian diet was an independent protective factor for incident GERD, with a significant protective effect observed in those who followed the diet for ≥6 years. However, the association between diet and the pathophysiological mechanism of GERD is unclear. Martinucci et al. [50] conducted a study of 165 participants who had negative endoscopy findings and heartburn and underwent impedance-pH monitoring while not receiving treatment. The participants were given two different meals: one primarily composed of animal protein and one primarily composed of vegetable protein. The results showed a significantly higher number of acid reflux episodes (7.5 ± 4.2 vs. 3.3 ± 2.8; *p* < 0.001), total number of reflux episodes (12.4 ± 9.9 vs. 6.3 ± 3.9; *p* < 0.001), and acid exposure time (3.3 ± 2.7% vs. 0.9 ± 1.4%; *p* = 0.005) in the first postprandial hour in those who received animal protein compared to those who received vegetable protein [50].

Several studies have also linked a vegetarian diet to a lower BMI. Wright et al. [51] performed a randomized controlled trial of 65 patients and classified them into low-fat plant-based diet and control groups. The results showed a significantly greater reduction in mean BMI in the plant-based group compared to the control group (4.4 vs. 0.4, difference: 3.9 kg/m^2^, *p* < 0.001) at 6 months [51,52,53]. Numerous studies have demonstrated a relationship between GERD and obesity. De Bortoli et al. [54] conducted a prospective open-label study of 116 patients and classified them into group A (reduced body weight by at least 10%) and group B (standard care). The results showed a significant decrease in perceived symptoms (*p* < 0.05) in both groups when receiving proton pump inhibitor therapy; however, the improvement was greater in Group A [7]. Moreover, a systematic review and meta-analysis of epidemiological studies examining associations among obesity with various disorders related to GERD found that the risk of erosive esophagitis and GERD symptoms was 1.5 to two times higher in obese individuals [54].

Several observational studies in India and Iran also suggested that a vegetarian diet could serve as a protective factor against GERD [18,19,20,21]. In a cross-sectional study conducted in Korea, Jung et al. [18] compared reflux esophagitis prevalence among 148 Buddhist monks, and found an association between non-vegetarianism with a higher risk of reflux esophagitis (odds ratio [OR] = 2.08; 95% CI: 1.086–3.974, *p* = 0.027) [18]. In addition, a systematic review in India found a pooled GERD prevalence of 15.6% (95% CI: 11.046 to 20.714), and that the risk factors were tobacco/alcohol consumption, drinking tea/coffee, age, a non-vegetarian diet, and BMI. However, significant heterogeneity was identified in the studies [19]. The Indian Society of Gastroenterology published evidence-based practice guidelines for the management of GERD in adults, in which spices and non-vegetarian food were the dietary factors associated with GERD (level of evidence: III, grade of recommendation: C) [20]. Moreover, a cross-sectional study in Iran involving 3846 individuals identified four major types of diet: vegetarian, traditional, Western, and fast food. While these dietary patterns were not significantly associated with the uninvestigated reflux risk, uninvestigated reflux was relatively positively associated with the fast food and traditional diets [21]. Furthermore, a survey of the general Italian population investigated the impact of a vegan (plant-only) diet on GERD [23]. Analysis of the 1077 participants found associations between following a vegan diet with a higher quality of life (all dimensions, *p* < 0.05) and reduced risk of GERD-related symptoms (OR = 0.47, 95% CI 0.28–0.81, *p* = 0.006).

Quality of life was assessed through self-reported measures, which included rating their health compared to 1 year ago, limitations in a series of activities, and how much emotional or physical health issues hindered normal family social activities [23]. Little research has focused on the associations between the duration and type of a vegetarian diet with GERD. A cross-sectional study conducted in India of 156,317 adults (20–49 years) assessed the associations between different types of vegetarian diet (vegan, lacto-, lacto-ovo, pesco-, semi-, and non-vegetarian) with BMI and self-reported diabetes. The results showed that those adhering to a pesco-vegetarian diet had the lowest mean BMI (20.3 kg/m²) followed by those adhering to a vegan diet (20.5 kg/m^2^), and that those adhering to a lacto-vegetarian diet had the highest mean BMI (21.2 kg/m^2^) followed by those adhering to a lacto-ovo vegetarian diet (21.0 kg/m^2^) [55]. Although the study found no significant differences in GERD incidence among the different types of vegetarian diets (vegan, lacto-, ovo-, lacto-ovo-vegetarian). However, the sample sizes for some subgroups (e.g., vegan, lacto-vegetarian) are relatively small, limiting the power to detect differences.

The findings of this study underscore the complex interplay between dietary factors and GERD. Notably, the negative association of vegetarian diets with GERD incidence aligns with previous research, which demonstrated that a lower consumption of meat and a higher intake of fruits and vegetables contribute to reduced GERD symptoms [56]. In addition, dietary habits such as midnight snacking and eating quickly have been identified as risk factors for GERD, further highlighting the importance of dietary choices. We also explored the potential physiological mechanisms underlying the protective effects of a vegetarian diet, which indicated that high-carbohydrate diets could exacerbate acid reflux symptoms [57]. In contrast, a vegetarian diet, typically lower in carbohydrates and rich in fiber, may promote gastrointestinal motility and balance the gut microbiome, potentially leading to lower reflux rates. Furthermore, our findings suggest that healthcare providers should consider recommending plant-based diets as part of a comprehensive strategy for managing GERD. This is particularly relevant in the context of diverse dietary practices, as cultural variations can significantly influence GERD risk factors, emphasizing the need for tailored dietary recommendations in different populations.

A vegetarian diet, rich in fiber and low in fat, can reduce pressure on the lower esophageal sphincter, preventing acid reflux. Fiber also promotes gastric emptying, reducing the time stomach contents stay in the stomach, which lowers the risk of acid regurgitation into the esophagus [58]. In addition, the antioxidant properties of plant-based foods may reduce inflammation in the esophagus and help repair mucosal damage [59]. Moreover, a vegetarian diet may also promote a healthier gut microbiota, which has been linked to a reduced risk of gastrointestinal disorders [60,61]. The inclusion of various phytochemicals, such as flavonoids and vitamins C and E, found in fruits and vegetables may also provide protective effects against oxidative stress, potentially reducing esophageal inflammation and damage [27,28,29]. Furthermore, the lower fat content associated with vegetarian diets may minimize the risk of gastroesophageal reflux, as high-fat meals are known to exacerbate reflux symptoms [56]. However, more randomized controlled trials are needed to fully understand these possible physiological mechanisms between vegetarian diets and lower GERD incidence.

Taken together, the aforementioned studies suggest that a vegetarian diet may decrease the occurrence of GERD due to lower rates of animal protein intake and obesity. Moreover, obesity has been linked with complications from longstanding reflux including esophageal adenocarcinoma, Barrett’s esophagus, and erosive esophagitis [62]. Furthermore, a large cohort study of 10,545 women found a dose-dependent relationship between higher BMI and frequent reflux symptoms (*p* for trend < 0.001) [63]. Given the association between a vegetarian diet and a lower incidence of GERD, health professionals could consider recommending plant-based diets as a non-pharmacological strategy in the management of patients with GERD or those at elevated risk of developing GERD. Promoting a vegetarian diet, particularly if followed long-term, could be part of a comprehensive approach to reducing the occurrence of symptoms and complications related to GERD.

The role of beverage choice in development of GERD symptoms is frequently discussed. The Nurses’ Health Study II cohort conducted 262,641 person-years of follow-up. No association was found between the consumption of milk, water, or juice and the risk of experiencing GERD symptoms. In a substitution analysis, replacing two servings per day of coffee, tea, or soda with two servings of water was associated with a reduced risk of GERD symptoms. The HRs were as follows: coffee HR = 0.96 (95% CI, 0.92–1.00); tea HR = 0.96 (95% CI, 0.92–1.00); and soda HR = 0.92 (95% CI, 0.89–0.96) [64]. At the cellular level, tissue-bound pepsin is fundamental to the pathophysiologic mechanism of reflux disease. pH 8.8 alkaline water irreversibly inactivated human pepsin (in vitro); it may have therapeutic benefits for patients with reflux disease [65]. The influence of fluid intake on GERD requires more randomized controlled trials and fundamental biological experiments.

Our research is based on a large community-based cohort in Taiwan, incorporating information on lifestyle, medical history, and environmental factors. The TWB offers an ideal platform for investigating both risk factors and protective factors associated with common diseases, including GERD. Nevertheless, there are also several limitations to this study. First, the presence of GERD was recorded according to questionnaire responses without verification of classic symptoms, and so data such as the type and severity of GERD were not available. The approach was prone to recall bias, and might not obtain the true incidence of GERD. Future studies using data from patient medical records or validated clinical assessments would enhance the ability to assess the accuracy of the diagnosis. Second, we lacked detailed information on the participants’ dietary patterns beyond their vegetarian status. The definition of “vegetarian” is broad and does not account for differences in dietary quality or nutrient intake. Future studies should incorporate more granular dietary data, such as food frequency questionnaires, fluid intake, or 24 h dietary recalls, to better understand the nutrient composition of the diets. Third, the dietary classification relied on self-declaration through a series of structured, closed-ended questions, which may introduce response bias, lack of nuance, misclassification, and temporal changes in dietary habits. Fourth, our study participants were recruited from the TWB, which adheres to specific guidelines in its questionnaire. According to these guidelines, an individual is classified as vegetarian only if they have followed this diet for at least six months. We had to comply with this criterion, and we acknowledge it as a limitation of our study. In addition, the study lacked several potential confounders related to GERD risk, such as the cooking practices, stress levels, sleep patterns, and the use of proton pump inhibitors or other medications. These factors could influence the incidence of GERD independently of dietary patterns. Lastly, considering the Chinese ethnicity of our participants, our findings may not be applicable to other groups.

## 5. Conclusions

In conclusion, the results of this study showed that following a vegetarian diet was an independent protective factor for incident GERD, with a significant protective effect observed in those who maintained a vegetarian diet for 6 years or more. Future studies should investigate the underlying mechanisms and explore whether adopting a vegetarian diet can reduce the incidence of GERD.

## Figures and Tables

**Table 1:** Baseline characteristics of the study subjects in the Taiwan Biobank cohort divided by vegetarian diet status.

Characteristics	All	Never	Ever	Current	*p*
	*n* = 23,714	*n* = 21,632	*n* = 930	*n* = 1152	
Age (years)	51 ± 10	51 ± 11	49 ± 10 *	52 ± 9 *^†^	<0.001
Body mass index (kg/m^2^)	24.1 ± 3.6	24.1 ± 3.6	24.0 ± 3.7 *	23.4 ± 3.6 ^†^	<0.001
Systolic blood pressure (mmHg)	118.1 ± 18.3	118.3 ± 18.3	116.0 ± 18.6 *	116.6 ± 18.1 *	<0.001
Diastolic blood pressure (mmHg)	72.7 ± 11.2	72.9 ± 11.2	71.8 ± 11.5 *	71.0 ± 11.0 *	<0.001
Heart rate (/30 s)	34.7 ± 4.6	34.7 ± 4.6	35.0 ± 4.7 *	35.3 ± 4.7	<0.001
Female, *n* (%)	15,316 (65)	13,812 (64)	656 (71) *	848 (74) *	<0.001
Ever married, *n* (%)	21,619 (91)	19,813 (92)	809 (87) *	997 (87) *	<0.001
Dependency, *n* (%)	1519 (6)	1324 (6)	90 (10) *	105 (9) *	<0.001
Ever smoking, *n* (%)	5700 (24)	5300 (25)	221 (24)	179 (16) *^†^	<0.001
Ever drinking alcohol, *n* (%)	1972 (8)	1858 (9)	65 (7)	49 (4) *^†^	<0.001
Exercise habits, *n* (%)	10,794 (46)	9962 (46)	373 (40) *	459 (40) *	<0.001
Educational status					<0.001
Elementary school, *n* (%)	1765 (7)	1582 (7)	53 (6)	130 (11) *^†^	
Middle to high school, *n* (%)	10,433 (44)	9525 (44)	373 (40) *	535 (47) *^†^	
University and above, *n* (%)	11,516 (49)	10,525 (49)	504 (54) *	487 (42) *^†^	
Hypertension, *n* (%)	2991 (13)	2788 (13)	89 (10) *	114 (10) *	<0.001
Diabetes, *n* (%)	1219 (5)	1131 (5)	48 (5)	40 (4)	0.031
Dyslipidemia, *n* (%)	1644 (7)	1542 (7)	53 (6)	49 (4) *^†^	<0.001
Depression, *n* (%)	705 (3)	618 (3)	47 (5) *	40 (4)	<0.001
Manic depression, *n* (%)	119 (0.5)	102 (0.5)	12 (1.3) *	5 (0.4) ^†^	0.002
Parkinson’s disease, *n* (%)	22 (0.1)	19 (0.1)	3 (0.3)	0 (0)	0.040
Chronic kidney disease, *n* (%)	369 (1.6)	341 (1.6)	18 (1.9)	10 (0.9)	0.106

* *p* value < 0.05 compared with never group; ^†^ *p* value < 0.05 compared with ever group. Abbreviation: *n* = number of participants.

**Table 2:** Univariable Cox regression for the risk of GERD.

Variables	HR (95% CI)	*p*
Age (per 1 year)	1.022 (1.018 to 1.027)	<0.001
Body mass index (per 1 kg/m^2^)	1.003 (0.990 to 1.016)	0.647
Systolic blood pressure (per 1 mmHg)	1.006 (1.003 to 1.008)	<0.001
Diastolic blood pressure (per 1 mmHg)	1.005 (1.001 to 1.009)	0.009
Heart rate (per 1 beat)	1.004 (0.994 to 1.014)	0.426
Female (vs. male)	0.802 (0.726 to 0.886)	<0.001
Ever married	1.291 (1.073 to 1.554)	0.007
Dependency	0.956 (0.785 to 1.164)	0.654
Ever smoking	0.993 (0.891 to 1.107)	0.899
Ever drinking alcohol	0.997 (0.841 to 1.182)	0.972
Exercise habits	1.109 (1.011 to 1.217)	<0.001
Educational status (middle to high vs. elementary school)	1.201 (1.001 to 1.441)	0.049
Educational status (university and above vs. elementary school)	1.066 (0.888 to 1.280)	0.490
Hypertension	1.405 (1.234 to 1.601)	<0.001
Diabetes	1.101 (0.891 to 1.359)	0.373
Dyslipidemia	1.556 (1.323 to 1.830)	<0.001
Depression	1.837 (1.486 to 2.270)	<0.001
Manic depression	1.695 (1.020 to 2.817)	0.042
Parkinson’s disease	0.935 (0.132 to 6.645)	0.947
Chronic kidney disease	0.993 (0.692 to 1.425)	0.970
Vegetarian diet status (Current vs. Never)	0.710 (0.557 to 0.905)	0.006
Vegetarian diet status (Ever vs. Never)	1.105 (0.875 to 1.394)	0.402

Values expressed as hazard ratio (HR) and 95% confidence interval (CI).

**Table 3:** Multivariate-adjusted hazard ratio for the GERD according to vegetarian diet status.

Vegetarian Status	GERD Cases/Subjects (*n*, %)	Adjusted HR (95% CI)	*p*
Never	1672/21,632 (7.7)	reference	-
Current	68/1152 (5.9)	0.697 (0.546–0.889)	0.004
Ever	74/930 (8.0)	1.086 (0.860–1.372)	0.489

Values expressed as hazard ratio (HR) and 95% confidence interval (CI). Covariates in the multivariable-adjusted model included age, body mass index, systolic and diastolic blood pressure, heart beats, sex, ever married, dependency, ever smoking, ever drinking alcohol, exercise habits, educational status, hypertension, diabetes, dyslipidemia, depression, manic depression, alcoholism, schizophrenia, Parkinson’s disease, chronic kidney disease, and vegetarian diet status.

**Table 4:** Multivariate-adjusted hazard ratios for the development of GERD according to years of vegetarian diet.

Years of Vegetarian Diet	GERD Cases/Subjects (*n*, %)	Adjusted HR (95% CI)	*p*
Never (0 years)	1672/21,632 (7.7)	reference	-
<6 years	78/1069 (7.3)	1.020 (0.812–1.281)	0.865
≥6 years	64/1013 (6.3)	0.717 (0.558–0.922)	0.009
*p* value for trend			0.033

Values expressed as hazard ratio (HR) and 95% confidence interval (CI). Covariates in the multivariable-adjusted model included age, body mass index, systolic and diastolic blood pressure, heart beats, sex, ever married, dependency, ever smoking, ever drinking alcohol, exercise habits, educational status, hypertension, diabetes, dyslipidemia, depression, manic depression, alcoholism, schizophrenia, Parkinson’s disease, chronic kidney disease, and years of vegetarian diet.

**Table 5:** Multivariate-adjusted hazard ratios for the development of GERD according to type of vegetarian diet.

Type of Vegetarian Diet	GERD Cases/Subjects (*n*, %)	Adjusted HR (95% CI)	*p*
Never	1672/21,632 (7.7)	reference	-
Vegan	15/239 (6.3)	0.792 (0.476–1.317)	0.368
Lacto-vegetarian	13/150 (8.7)	0.986 (0.571–1.704)	0.961
Ovo-vegetarian	8/105 (7.6)	0.936 (0.467–1.879)	0.853
Lacto-ovo-vegetarian	106/1588 (6.7)	0.848 (0.697–1.033)	0.102

Values expressed as hazard ratio (HR) and 95% confidence interval (CI). Covariates in the multivariable-adjusted model included age, body mass index, systolic and diastolic blood pressure, heart beats, sex, ever married, dependency, ever smoking, ever drinking alcohol, exercise habits, educational status, hypertension, diabetes, dyslipidemia, depression, manic depression, alcoholism, schizophrenia, Parkinson’s disease, chronic kidney disease, and type of vegetarian diet.

## Data Availability

The data underlying this study are from the Taiwan Biobank. Due to restrictions placed on the data by the Personal Information Protection Act of Taiwan, the minimal data set cannot be made publicly available. Data may be available upon request to interested researchers. Please send data requests to: Szu-Chia Chen, PhD, MD. Division of Nephrology, Department of Internal Medicine, Kaohsiung Medical University Hospital, Kaohsiung Medical University.
